# IDCC-SAM: A Zero-Shot Approach for Cell Counting in Immunocytochemistry Dataset Using the Segment Anything Model

**DOI:** 10.3390/bioengineering12020184

**Published:** 2025-02-14

**Authors:** Samuel Fanijo, Ali Jannesari, Julie Dickerson

**Affiliations:** 1 Department of Computer Science, Iowa State University, Ames, IA 50010, USA; jannesar@iastate.edu; 2 Department of Electrical and Computer Engineering, Iowa State University, Ames, IA 50010, USA

**Keywords:** cell counting, biomedical data processing, artificial intelligence, large foundation models, fluorescence microscopy, cellular biology, applied computing

## Abstract

Cell counting in immunocytochemistry is vital for biomedical research, supporting the diagnosis and treatment of diseases such as neurological disorders, autoimmune conditions, and cancer. However, traditional counting methods are manual, time-consuming, and error-prone, while deep learning solutions require costly labeled datasets, limiting scalability. We introduce the Immunocytochemistry Dataset Cell Counting with Segment Anything Model (IDCC-SAM), a novel application of the Segment Anything Model (SAM), designed to adapt the model for zero-shot-based cell counting in fluorescent microscopic immunocytochemistry datasets. IDCC-SAM leverages Meta AI’s SAM, pre-trained on 11 million images, to eliminate the need for annotations, enhancing scalability and efficiency. Evaluated on three public datasets (IDCIA, ADC, and VGG), IDCC-SAM achieved the lowest Mean Absolute Error (26, 28, 52) on VGG and ADC and the highest Acceptable Absolute Error (28%, 26%, 33%) across all datasets, outperforming state-of-the-art supervised models like U-Net and Mask R-CNN, as well as zero-shot benchmarks like NP-SAM and SAM4Organoid. These results demonstrate IDCC-SAM’s potential to improve cell-counting accuracy while reducing reliance on specialized models and manual annotations.

## 1. Introduction

**Figure 1:**
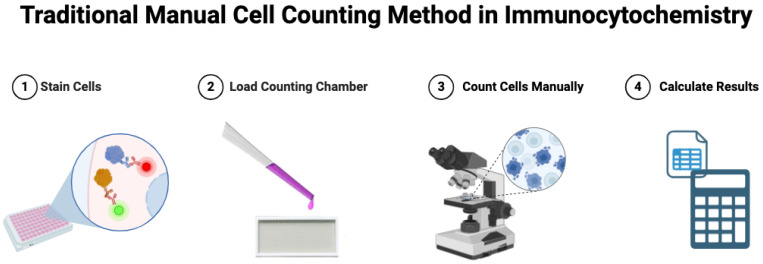
Traditional cell counting method in immunocytochemistry images. An outline of the traditional approach to cell counting using fluorescence microscopy for immunocytochemistry datasets. The method leverages fluorescence labeling to visualize cellular components and identify specific cell types or biomarkers within the sample. By manually counting the fluorescently labeled cells under a microscope, researchers can quantify cell populations and assess changes in cellular morphology or distribution for various applications, including disease diagnosis. However, it is time-consuming, error-prone, and often irreproducible. Created with BioRender.com.

Cell counting is crucial in many biomedical applications. However, traditional manual methods are labor-intensive, time-consuming, and prone to human error. Traditionally, experts use a microscope (Figure 1) to count cells [1], which is challenging to standardize. Distinguishing cells of varying illuminations, sizes, shapes, and densities using manual methods is an arduous task that can lead to inaccurate counts [2]. Automated cell counting techniques have emerged to mitigate these challenges, particularly machine and deep learning-based methods.

Machine learning methods leverage statistical models to classify and count cells, necessitating extensive feature engineering and parameter tuning, which makes them computationally expensive and time-consuming [3,4]. Deep learning-based methods, utilizing convolutional neural networks (CNNs) with limited parameter tuning and multiple layers have shown superior performance in various domains, including medical image analysis. Recent studies such as [5,6] have used deep learning to automate cell counting tasks in fluorescence microscopy and optical tissue images, achieving promising results. Instance segmentation methods, such as Mask R-CNN and U-NET, have also been utilized for blood cell counting and categorization [7,8], demonstrating competitive performance in detecting various types of blood cells.

**Research gap:** The performance of deep learning models in cell counting tasks is impressive, with the capacity to achieve high accuracy and efficiency. However, one significant limitation is the heavy reliance on annotated data, where acquiring such data can be expensive and time-consuming. The cost and effort required to label large datasets manually hinder the scalability and accessibility of these models, particularly in research settings with limited resources.

**IDCC-SAM’s contributions:** To overcome these challenges and make automated cell counting more accessible and cost-effective, there is a growing need for zero-shot models. Zero-shot capability is the ability of a model to generalize to unseen tasks or domains without explicit task-specific supervision or training. By leveraging pre-trained representations, zero-shot models like IDCC-SAM offer a promising solution for automating cell counting tasks while mitigating the burden of data annotation and high computational training resources.

IDCC-SAM’s backbone is built on the Segment Anything Model (SAM), developed by Meta [9], which has emerged as one of the advanced pre-trained models, also called foundation models, in literature for segmentation tasks, and which can precisely segment or separate arbitrary objects from their background in natural images. Our approach employs SAM to operate under zero-shot, unprompted conditions, demonstrating its capability to achieve good results without the requirement for labeled data or training sets. Therefore, we leverage its segmentation ability for cell counting. To validate the generalizability and effectiveness of our proposed method, we evaluate it on three (3) public benchmarks for cell counting—the immunocytochemistry dataset for cellular image analysis (IDCIA) [10], human subcutaneous adipose tissue (ADC) dataset [11], and VGG synthetic fluorescence microscopy dataset [12].

**Target audience:** This research is intended primarily for cellular biologists and researchers working in cell counting tasks, particularly in the context of immunocytochemistry. Additionally, the tool may benefit adjacent fields such as histopathology, flow cytometry, and high-content imaging, where automated cell segmentation and counting are essential. By providing a scalable and automated solution, this work aims to assist researchers in both academic and clinical settings, especially those facing challenges with large-scale manual annotation.

In summary, the main contributions of this work are as follows:

**(1) A new end-to-end zero-shot pipeline for automated cell counting** for fluorescent microscopic immunocytochemistry images. To the best of our knowledge, this is the first zero-shot applied implementation of the Segment Anything Model (SAM) on an immunocytochemistry dataset.

**(2) Advanced state-of-the-art performance**: IDCC-SAM achieves superior results compared with existing cell counting methods on multiple datasets, setting a new baseline for zero-shot immunocytochemistry cell counting performance, and providing a foundation for future research in this domain.

The remainder of this paper is structured as follows. In Section 2, we review existing approaches to cell counting, categorizing them into image-processing, density-based, and segmentation-based methods. In Section 3, we describe the proposed IDCC-SAM pipeline and its components in detail, the datasets, and metrics that we used in evaluating IDCC-SAM. Section 4 presents the evaluation of IDCC-SAM on three public datasets and compares its performance with four (4) benchmark methods. Finally, Section 5 and Section 6 summarize our contributions and outline limitations, as well as directions for further improvement.

## 2. Related Works

Cell counting is vital in the field of biomedical research and healthcare, enabling doctors and medical professionals to diagnose, monitor, and treat a wide range of disease phenotypes [13] such as anemia, autoimmune disorders, leukemia, sickle cell, and cancer. With advancements in imaging and computational techniques, researchers have proposed several approaches, broadly categorized into image-processing-based, density-based, and segmentation-based methods.

**Image processing-based methods:**

Traditional image processing techniques have been widely applied to cell counting tasks, leveraging methods such as background removal, blob detection, and edge detection to isolate and count cells [14,15,16,17]. For example, Biswas et al. [16] utilized watershed segmentation to distinguish cell boundaries, while Jiang et al. [17] proposed a watershed clustering-based approach for cell isolation. While these methods are computationally efficient and simple, their effectiveness is limited in the presence of overlapping cells, noisy data, or inconsistent lighting conditions.

Recent work has focused on improving these methods with adaptive algorithms for noise reduction and edge detection, particularly in noisy or low-resolution images. For example, ref. [18] introduced a novel adaptive canny edge detection technique using fast median filtering, achieving improved performance in challenging environments, while [19] also developed an enhanced edge detection method using Laplacian Gaussian filtering from denoising images. A summary of these related works, including key challenges and advantages, is shown in Table 1.

**Density-based methods:**

Density-based methods have gained traction by employing feature-based regression to estimate cell counts. These approaches typically involve segmenting dense regions, extracting features such as area or texture, and applying regression functions to predict counts [21,22].

For instance, ref. [23] developed a single-image crowd-counting method using a multi-column convolutional neural network. Following that, ref. [22] introduced a fully convolutional regression network to perform cell counting in microscopy images, while [21] developed a dilated convolutional neural network to count cells in highly congested scenes. More recently, ref. [20] integrated transformer-based encoders with density-based regression models, enhancing feature extraction and cell counting for underwater fish counting. Despite their advantages in handling varying densities, these methods often require substantial computational resources, careful parameter tuning, and diverse training datasets to mitigate overfitting.

**Segmentation-based methods:**

Deep learning models have facilitated significant progress in cell counting by enabling the direct learning of features from annotated datasets. Pioneering models such as U-Net [24] and Mask R-CNN [25] have demonstrated remarkable accuracy in segmenting individual cells in complex images [30,31]. Recent advances include [26], where the authors proposed a multi-transformer U-Net variant that improved segmentation accuracy, particularly for challenging structures in medical images. However, these methods, while highly accurate and scalable, are heavily dependent on the availability of large, annotated datasets, which are often expensive and time-consuming to generate.

**Foundation models:**

Pre-trained (foundation) models have demonstrated strong adaptability across various tasks without explicit fine-tuning [27,32,33]. More particularly in computer vision, they have excelled in several downstream segmentation tasks with minimal modifications, gaining an advantage over traditional approaches that may require extensive parameter tuning.

Various studies have shown SAM’s superior efficacy in the medical domain compared with supervised settings [28,29,34]. In this work, we specifically demonstrate its application in immunocytochemistry cell counting.

## 3. Materials and Methods

We present a novel application of the Segment Anything Model (SAM), IDCC-SAM (Immunocytochemistry Dataset Cell Counting with SAM), designed to adapt the Segment Anything Model for zero-shot-based cell counting in fluorescent microscopic immunocytochemistry images datasets. IDCC-SAM is an end-to-end automated pipeline consisting of three key components: preprocessing, mask extraction, and post-processing. Each stage plays a critical role in transforming raw cellular images into accurate cell counts.

First, the preprocessing stage enhances the illumination of cellular images using a technique called contrast-limited adaptive histogram equalization (CLAHE). This method adjusts the image contrast to correct uneven lighting, ensuring that the images are well-suited for subsequent analysis.

Next, in the mask extraction stage, IDCC-SAM segments the cellular images into distinct regions (masks) representing individual cells by leveraging the pre-trained image encoder and mask decoder in SAM to identify and separate cells from the background without requiring additional training.

Finally, we introduce a post-processing technique that refines the segmentation masks and identifies the center points of the cells, enabling accurate cell counting. This stage transforms the segmented regions into actionable data for downstream quantitative analysis.

In the following sections, we first briefly review the architecture of SAM on which IDCC-SAM is built, followed by specific modifications and enhancements introduced in our method. Finally, we describe the dataset that we use, as well as our evaluation metrics for IDCC-SAM.

### 3.1. Preliminaries: Segment Anything Model (SAM)

The Segment Anything Model (SAM) [9] comprises three primary modules. **(a)** The image encoder, leveraging a vision transformer (ViT)-based backbone for extracting features from images, producing an image embedding. **(b)** The prompt encoder, responsible for encoding positional information derived from input points, boxes, or masks to facilitate the mask decoder’s operation. **(c)** The mask decoder, employing a multi-layer transformer-based decoder that integrates the extracted image embeddings with any concatenated output tokens to produce final mask predictions.

SAM is trained on the large SA-1B dataset, which consists of 11M diverse, high-resolution, licensed, and privacy-protecting images and 1.1B masks (pixel-level annotations that delineate distinct objects or regions within an image) [9]—making it the largest segmentation dataset currently available. This extensive training facilitates SAM’s zero-shot generalization to new data without requiring additional training. SAM’s training process is resource-intensive, with distributed training of the ViT-H-based SAM for just 2 epochs on SA-1B, requiring 256 GPUs and a large batch size of 256 images. For a detailed description of SAM, please refer to original paper [9].

### 3.2. Ours: IDCC-SAM

**Figure 2:**
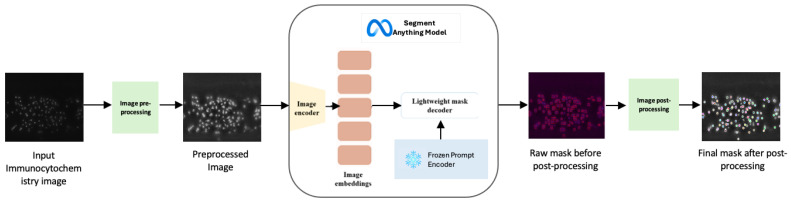
Overview of IDCC-SAM—beginning with the input immunocytochemistry image, which undergoes image preprocessing using CLAHE to enhance illumination. The preprocessed image is then fed into the Segment Anything Model (SAM), where the image encoder converts it into high-dimensional feature embeddings. These embeddings are processed by the lightweight mask decoder to generate raw segmentation masks. Finally, image post-processing is applied to refine the masks and perform cell counting. The prompt encoder is frozen as IDCC-SAM is designed as an automated end-to-end pipeline without human-in-the-loop.

Our modification of SAM to become IDCC-SAM consists of three major stages, as shown in Figure 2: **(a)** a preprocessing step using contrast limited adaptive histogram equalization (CLAHE) [35]; **(b)** the extraction of masks using SAM, which separates cells from the background; and **(c)** post-processing of masks to identify the center points and perform cell counting.

#### 3.2.1. Preprocessing

For the first stage, to improve the uneven illumination of the immunocytochemistry cellular images, we apply contrast-limited adaptive histogram equalization (CLAHE) [35] to all images before feeding it into stage two, which is the main IDCC-SAM architecture. This happens through a multi-stage process. Initially, each input image is divided into non-overlapping 8 × 8 tiles, each representing a neighborhood of 64 pixels. An intensity histogram is then computed for each tile to capture the distribution of pixel intensities within that region. To prevent over-amplification of noise, clip limits are set, with a threshold parameter of 0.8 producing the best illumination for IDCC-SAM. This clipping ensures that the intensity values do not exceed a certain maximum, maintaining a balanced enhancement across the image.

Using these clip limits, the intensity values within each tile are normalized locally through a cumulative probability distribution function. This function adjusts the pixel values based on the frequency of intensity levels in the histogram, effectively distributing the intensity values more evenly across the image. Finally, the neighboring tiles are combined and remapped using bilinear interpolation. This step enhances the overall image grayscale, producing a more evenly illuminated image that is suitable for further analysis. This preprocessing stage is crucial for improving the visibility and quality of the immunocytochemistry cellular images, making them ready for the subsequent stages in the IDCC-SAM pipeline.

Formally, our implementation of the CLAHE preprocessing stage is described in Algorithm 1:
**Algorithm 1** IDCC-SAM CLAHE Preprocessing Stage1:**Step 1**: Divide each input image into nonoverlapping 8×8 tiles. Each block corresponds to a neighborhood of 64 pixels.2:**Step 2**: Compute the intensity histogram of each region.3:**Step 3**: Set the clip limits, *c*, for clipping the histograms. For IDCC-SAM, c=0.8 produced the best illumination.4:**Step 4**: Using the clip limit in Step 3, normalize intensity values locally using a cumulative probability distribution (CPD) function, given as:(1)$$p=\left[p_{\max}-p_{\min}\right]*P\left(f\right)+g_{\min}$$**Where:**$p_{\max}$ = Maximum pixel value$p_{\min}$ = Minimum pixel value$g_{\min}$ = Minimum grayscale value in the normalized range*p* is the computed pixel valueP(f) = CPD (Cumulative Probability Distribution), which can also be formally written as:
(2)$P\left(f\right)=\frac{\sum_{i=0}^{f}H\left(i\right)}{\sum_{i=0}^{L-1}H\left(i\right)}$**Where:**H(i) is the frequency of intensity level *i* in the histogram.*f* is the intensity value up to which the histogram values are summed.*L* is the number of intensity levels.5:**Step 5**: Combine and remap each neighboring tile using bilinear interpolation and enhance image grayscale using the modified histograms.

#### 3.2.2. Extraction of Cell Masks

In the second stage of the IDCC-SAM pipeline, we focus on the extraction of masks, which involves processing the input images through four (4) key sub-components of the Segment Anything Model (SAM), as illustrated in the overview in Figure 2.

1. **Image encoder:** the process begins with the image encoder, which transforms the input cellular image into a set of high-dimensional feature embeddings. The image encoder in SAM is based on the huge vision transformer (ViT-H) architecture. The input image is divided into fixed-size patches of 16 × 16 pixels. Each patch is then linearly embedded into a 1D vector, resulting in a sequence of patch embeddings. These embeddings are processed through 32 transformer blocks, which capture both local and global contextual information. The output of the image encoder is a sequence of image embeddings, each representing the features of a 16 × 16 patch of the original cellular image. The size of each embedding is 768, resulting in an output tensor of size $R^{d_{i}\times \frac{W}{16}\times \frac{H}{16}}$, where $d_{i}=768$.
2. **Image embeddings:** the image embeddings are the outputs of the ViT encoder and serve as a compact representation of the input image. These embeddings have a lower spatial resolution compared with the input image, specifically $\frac{H}{16}\times \frac{W}{16}$, where *H* and *W* are the height and width of the input image. Each embedding captures the salient features necessary for accurate identification of the cellular features. In the zero-shot setting, these embeddings are generated without any additional training or fine-tuning on the target dataset, relying solely on the pre-trained knowledge (from the large SA-1B dataset) of the SAM model.
3. **Lightweight mask decoder:** the lightweight mask decoder receives the image embeddings from the ViT encoder, and is responsible for transforming these embeddings into cell masks (segmentations). The mask decoder consists of:
  - **Convolutional layers:** these layers refine the feature maps from the image embeddings, enhancing their spatial resolution and capturing low-level details.
  - **Upsampling operations:** these operations increase the spatial resolution of the feature maps to match the original image dimensions.
  - **Attention mechanisms:** they enable the model to focus on the most relevant parts of the feature maps, ensuring accurate delineation of the cellular structures that exist in the input image.
  The mask decoder combines these operations to generate high-resolution masks that accurately segment cellular structures. The lightweight nature of the decoder ensures that the mask generation process is computationally efficient.
4. **Prompt encoder (frozen):** although the prompt encoder is a part of the SAM architecture, it is not utilized in the IDCC-SAM pipeline for the extraction of masks. The prompt encoder typically processes user-provided prompts, such as points, boxes, or masks, to refine the segmentation output. In our zero-shot approach, we bypass the prompt encoder, allowing the IDCC-SAM to generate segmentation masks autonomously based on the image embeddings alone, eliminating the need for manual annotations or human-in-the-loop. Since the prompt encoder in SAM utilizes a default embedding when there is no prompt provided, IDCC-SAM retains this default embedding.

Using these components, the IDCC-SAM pipeline effectively segments and counts cells in immunocytochemistry images without requiring manual intervention. The ViT-based image encoder provides feature extraction, while the lightweight mask decoder carries out mask generation. This automated approach significantly streamlines the workflow, enhancing the overall efficiency of the cell counting process.

#### 3.2.3. Post-Processing and Cell Counting

The final stage of the IDCC-SAM architecture involves post-processing the masks generated in the second stage to identify the center points of segmented cellular structures in order to perform cell counting. Because SAM treats the background of the image as a mask, the first post-processing step at this stage is to exclude this from the final masks. Furthermore, this stage consists of iterating through the segmented masks, calculating the center points of each segmented cellular object, counting the cells, and plotting them on the original image using markers. This approach facilitates easier visualization and analysis of the identified cell structures, aiding further downstream analysis and interpretation of the cell counting results.

Formally, let $M=\{m_{0},m_{1},m_{2},\ldots ,m_{n}\}$ represent the set of cell masks generated in the second stage, where *n* is the number of masks. For each mask, $m_{i}$, we compute the bounding box, $\mathrm{bbox}(m_{i})$, defined by its coordinates $\left(x_{i},y_{i},w_{i},h_{i}\right)$, where $x_{i}$ and $y_{i}$ are the coordinates of the top-left corner, and $w_{i}$ and $h_{i}$ are the width and height of the bounding box, respectively. The bounding box is computed by finding the minimum and maximum *x* and *y* coordinates of the non-zero pixels in the mask. The center points are then computed using these coordinates and plotted on the original image to visualize its segmented cellular structures, with each mask assigned a unique color.

The complete post-processing and cell counting algorithm is formally shown in Algorithm 2.

Overall, the introduction of this post-processing method improves the accuracy of the cell counting and enhances the interpretability of the results by providing a clear and distinct visualization of each segmented cell on the original image. Furthermore, its automated approach streamlines the workflow and significantly enhances the efficiency of the cell counting task.
**Algorithm 2** IDCC-SAM Post-processing and Cell Counting Algorithm  1:Initialize an empty list *center_points* to store the center points of the segmented masks.  2:Skip the first mask $m_{0}$, which is the background (as SAM treats the background as the first mask).  3:**for** each mask $m_{i}\in \left\{m_{1},m_{2},\ldots ,m_{n}\right\}$ in *M* **do**  4:    Compute the bounding box coordinates $\left(x_{i},y_{i},w_{i},h_{i}\right)$:  5:    Find the non-zero pixel coordinates (x,y) in the mask $m_{i}$.  6:    Calculate the minimum and maximum coordinates:(3)$$x_{i}=\min\left(x\right),\;y_{i}=\min\left(y\right)$$(4)$$w_{i}=\max\left(x\right)-x_{i},\;h_{i}=\max\left(y\right)-y_{i}$$  7:    Calculate the center point $\left(c_{x_{i}},c_{y_{i}}\right)$ using:(5)$$c_{x_{i}}=x_{i}+\frac{w_{i}}{2}$$(6)$$c_{y_{i}}=y_{i}+\frac{h_{i}}{2}$$  8:    Append the center point $\left(c_{x_{i}},c_{y_{i}}\right)$ to *center_points*.  9:**end for**10:Plot the original image and overlay the center points using unique colors for each mask.11:Count the total number of center points to determine the number of cells *N*.

### 3.3. Datasets

**Figure 3:**
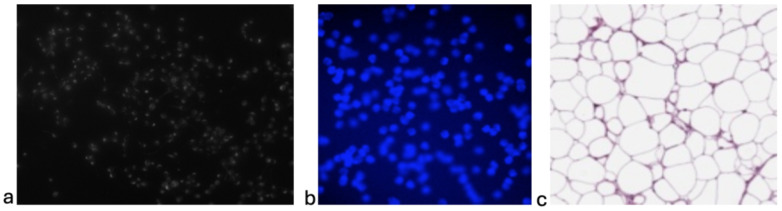
Sample images from each dataset. (**a**) Sample image from IDCIA; (**b**) sample image from VGG; (**c**) sample image from ADC.

To validate the effectiveness and generalizability of IDCC-SAM, we evaluate it on three (3) public benchmark datasets for immunocytochemistry cell counting, starting with the immunocytochemistry dataset for cellular image analysis (IDCIA) [36], the human subcutaneous adipose tissue (ADC) dataset [37], and the VGG synthetic fluorescence microscopy dataset [38]. Table 2 compares the datasets, while a sample image from each dataset is shown in Figure 3.

1. **IDCIA [36]:** the immunocytochemistry dataset for cellular image analysis (IDCIA) is a public dataset that was released in June 2023 by [10] as part of a study investigating the potential of electrical stimulation to modulate stem cell differentiation and possible applications for neural repair. The dataset contains 262 images (800 × 600 dimensions) of annotated neural cells of rat Adult Hippocampal Progenitor Cells (AHPCs) after electrical stimulation. The dataset includes images from experiments covering the use of seven primary antibodies, making it the most diverse immunocytochemistry dataset currently available. The average cell count per image is 83 ± 104.
2. **VGG [38]:** VGG is one of the first standard benchmark datasets for cell counting released by [12] in 2010. The dataset contains 200 images (256 × 256 dimension) of annotated simulated bacterial cells from fluorescence light microscopy. Each image contains 174 ± 64 cells at various focal distances, simulating real-life imaging with a microscope.
3. **ADC [37]:** ADC is a human subcutaneous adipose tissue dataset obtained from the Genotype-Tissue Expression Consortium [11]. It contains a total of 200 regions of interest (ROI) sampled from high-resolution histology slides, with each ROI representing adipocyte cellular images. The average cell count per image is 165 ± 44.

### 3.4. Evaluation Metrics

To assess the zero-shot capability of IDCC-SAM on the cell counting datasets, two quantitative metrics were employed: Mean Absolute Error (MAE) and Acceptable Absolute Error (AAE) Count Percentage. Mean Absolute Error is a gold standard for evaluating accuracy in cell counting tasks. It provides a straightforward assessment of model performance by measuring the average magnitude of errors between predicted and ground truth cell counts, without considering the direction of errors.

The AAE metric measures the percentage of images whose predicted count is within a difference of ±T of the true count by the domain expert, as shown in Equation (8), where *T* is a positive number. To complement the MAE metric, the AAE metric is more robust to imbalances in cell distribution, provides a more nuanced evaluation of the model, and offers a more intuitive interpretation and task-specific relevance.

Specifically, the MAE and AAE are calculated as:

(7) $$\mathrm{MAE}=\frac{1}{N}\sum_{i=1}^{N}\left|C_{i}-{\hat{C}}_{i}\right|$$

(8) $$\mathrm{AAE}=\frac{1}{N}\sum_{i=1}^{N}〚\left|C_{i}-{\hat{C}}_{i}\right|\leq T〛\times 100\%$$

where *N* is the total number of images, $C_{i}$ is the true cell count for image *i*, ${\hat{C}}_{i}$ is the predicted cell count for image *i*, and *T* is the best-performing threshold chosen based on empirical experiments in consultation with domain experts. The 〚.〛 denotes the Iverson brackets, which return 1 if the condition is satisfied and 0 otherwise. Based on domain experts’ input and experiments conducted under zero-shot settings, a threshold of 10 was chosen for the AAE evaluation in the final results.

A higher AAE indicates a better model, while a lesser MAE indicates a better model.

### 3.5. Comparing Methods

We compare IDCC-SAM with four (4) total benchmark methods—two (2) supervised methods, UNet [24] and Mask RCNN [25], as well as two (2) previous zero-shot methods on cell counting, SAM4Organoid [39] and NP-SAM [40]. These comparisons highlight the efficacy and generalizability of IDCC-SAM, compared with existing methods.

1. **UNet [24]:** UNet has proven in the literature to be highly effective for semantic segmentation and cell counting tasks [8,24], due to its ability to learn from a relatively small number of annotated data, making it effective for supervised learning tasks. The model is fine-tuned on 75% of each of the datasets, while the remaining 25% is used for evaluation. The details of the experimental setup used for fine-tuning the UNet model are provided in the Supplementary Materials.
2. **Mask RCNN [25]:** Mask RCNN, also a widely used instance segmentation model [41,42], extends Faster R-CNN by adding a branch for predicting segmentation masks on each region of interest (RoI), alongside the existing branch for classification. Similar to the U-Net training setup, the model is fine-tuned on 75% of each of the datasets, while the remaining 25% is used for evaluation. The details of the experimental setup used for fine-tuning are also provided in the Supplementary Materials.
3. **SAM4Organoid [39]:** SAM4Organoid is a zero-shot segmentation model designed specifically for organoid microscopy cellular images. Similar to how IDCC-SAM works, it also leverages the pre-trained Segment Anything Model (SAM) to perform cell segmentation and counting without training on the labeled dataset.
4. **Nano-Particle-SAM [40]:** Nano-Particle-SAM, also known as NP-SAM, implements the Segment Anything Model for cell segmentation in electron microscopy cellular images. Similar to SAM4Organoid and IDCC-SAM, NP-SAM operates in a zero-shot manner, utilizing SAM’s pre-trained capabilities to segment and count cells without a need for supervised training.

## 4. Results and Discussion

### 4.1. Standalone Results of IDCC-SAM—Overview

We evaluated IDCC-SAM’s performance across three datasets—VGG, ADC, and IDCIA—by analyzing its Mean Absolute Error (MAE) and Absolute Acceptable Error (AAE) in images with low and high cell densities (using 100 cells as the threshold), where a perfect MAE is 0, indicating no error, and a perfect AAE is 100%, reflecting complete agreement with ground truth. Table 3 provides a numerical summary of the results.

Overall, IDCC-SAM achieved its best performance on the ADC dataset, with the lowest MAE values for both low-density (8) and high-density (29) images, alongside a perfect AAE of 100% for low-density images. In comparison, the model performed moderately well on the VGG dataset, with an MAE of 11 (low density) and 30 (high density), and AAE values of 44% and 23%, respectively. However, IDCC-SAM exhibited the highest MAE and lowest AAE on the IDCIA dataset, with MAE values of 37 (low density) and 90 (high density), and AAE values of 40% and 14%, which can be attributed to the higher diversity in staining techniques in this particular dataset.

The next section discusses the results in more detail, focusing on IDCC-SAM’s performance relative to the datasets’ unique properties and the potential areas for improvement.

### 4.2. Standalone Results of IDCC-SAM—Discussion

As shown in Table 3, the performance of IDCC-SAM on the three datasets—VGG, ADC, and IDCIA—reveals distinct strengths and weaknesses based on the variation in cell densities, as well as the image properties of the cellular images. In the VGG dataset, IDCC-SAM demonstrates strong performance, particularly in low-density scenarios. The model achieves the lowest MAE (11) for low-density images and a competitive MAE (30) for high-density images. The AAE results reinforce this performance, with 44% for low-density and 23% for high-density images. This indicates that the model can effectively handle the variations in cell densities in this dataset, likely due to the relatively consistent staining and imaging conditions of the VGG dataset.

The ADC dataset is quite unique from the other datasets due to its (unconventional) bright white background compared with the others’ unevenly illuminated versions. IDCC-SAM gains an advantage from this, showing remarkable performance with the lowest MAE for both low-density images (8) and high-density images (29) across all three datasets. The AAE results are particularly impressive for low-density images, achieving 100%, indicating the model’s robustness in simpler, less crowded scenarios. However, for high-density images, the AAE drops to 23%, suggesting that, despite the bright background, the model is challenged by crowded cell areas.

In the IDCIA dataset, IDCC-SAM exhibits the highest Mean Absolute Error (MAE) for both low-density (37) and high-density (90) cell images, indicating a lower performance compared with the other datasets. The Absolute Acceptable Error (AAE) margin further highlights this issue, with the model achieving 40% for low-density images and only 14% for high-density images. This suggests that IDCC-SAM struggles significantly with the IDCIA dataset.

IDCC-SAM’s lowest performance with IDCIA can be attributed to several factors. First, IDCIA is characterized by diverse and complex cellular structures and staining methods (seven in total), making it more challenging for a zero-shot approach like IDCC-SAM. The variety in staining techniques introduces significant variability in the appearance of cells, which introduces varying challenges in the cell counting process. Additionally, the high density of cells can lead to overlapping structures that are difficult to separate accurately without fine-tuning the model on this specific dataset.

Second, the IDCIA dataset includes images with a wide range of brightness and contrast levels, which can further hinder the model’s performance. The preprocessing step using contrast-limited adaptive histogram equalization (CLAHE) helps to some extent, but the inherent variability in the dataset remains a significant challenge for IDCC-SAM.

In contrast, the VGG dataset, with its more uniform staining and imaging conditions, presents a less complex cell counting task, allowing IDCC-SAM to perform more effectively. Similarly, the ADC dataset, with its relatively brighter background and less variability in terms of staining techniques, aids in achieving better results.

Overall, the analysis shows that IDCC-SAM is highly effective in handling datasets with lower cell densities, particularly excelling in the ADC dataset. However, its performance in higher-density scenarios and images with significantly higher variability in staining methods, especially in the IDCIA dataset, provides opportunities for improvement in our future work.

### 4.3. Comparison with Benchmark Methods

To further demonstrate the efficacy of IDCC-SAM, we compared IDCC-SAM with four benchmark methods—UNet, Mask RCNN, SAM4Organoid, and NP-SAM—across three datasets: VGG, ADC, and IDCIA. For better clarity, the analysis is presented in two parts: (1) benchmark comparison per dataset, as shown in Table 4, Table 5 and Table 6, and (2) overall average performance across all datasets, summarized in Table 7.

In the per-dataset comparison, IDCC-SAM achieved the lowest MAE in the VGG and ADC datasets. For VGG (Table 4), IDCC-SAM excelled in low-density images (MAE: 11; AAE: 44%) and performed well in high-density images (MAE: 30; AAE: 23%), outperforming all benchmarks in both cases. On the ADC dataset (Table 5), IDCC-SAM showed exceptional accuracy in low-density images (MAE: 8; AAE: 100%) and retained its lead in high-density images (MAE: 29; AAE: 23%). For the IDCIA dataset (Table 6), IDCC-SAM achieved the highest AAE for low-density images (40%), matching UNet, but struggled with high-density images (MAE: 90), where Mask RCNN (64) and NP-SAM (77) performed better.

In the overall comparison across all datasets (Table 7), IDCC-SAM demonstrated strong performance, achieving the lowest average MAE for VGG (26) and ADC (28), with only a slight decline on IDCIA (MAE: 52, compared with UNet’s 43 and NP-SAM’s 47). Importantly, IDCC-SAM consistently achieved the highest overall AAE for all datasets: 28% (VGG), 26% (ADC), and 33% (IDCIA). These results highlight IDCC-SAM’s robustness in generalizing across different datasets and its ability to handle both low- and high-density cell images effectively.

Overall, IDCC-SAM’s superior performance in lower-density scenarios highlights its strength in accurately segmenting and counting cells when they are more dispersed. This is due to its effective mask generation (inherited from SAM [9]) and the post-processing technique we introduced, which allows for more precise cell identification and counting. However, the decline in performance with higher cell densities shows limitations in handling overlapping and occluded cells, which become more prominent in densely packed cellular images, particularly for a dataset like IDCIA. The fixed patch size and resolution of the ViT encoder in IDCC-SAM may contribute to losing essential details needed for distinguishing closely packed cells. Furthermore, the absence of fine-tuning with high-density training data—a benefit seen in models like UNet and Mask RCNN—also justifies why they may perform better than IDCC-SAM in some datasets. To address these challenges, our future work will explore incorporating few-shot fine-tuning, adjusting the ViT encoder’s resolution and patch sizes to become dynamic to different cell densities, and integrating domain-adaptive mask decoders into the IDCC-SAM architecture (while still limiting the need for large labeled datasets or computationally expensive training).

In summary, while IDCC-SAM outperformed benchmark methods in most scenarios, its performance on IDCIA highlights the challenges posed by datasets with high variability in staining techniques and densely packed cells. Future improvements will focus on addressing these limitations through fine-tuning and architectural adjustments.

### 4.4. Qualitative Results

**Figure 4:**
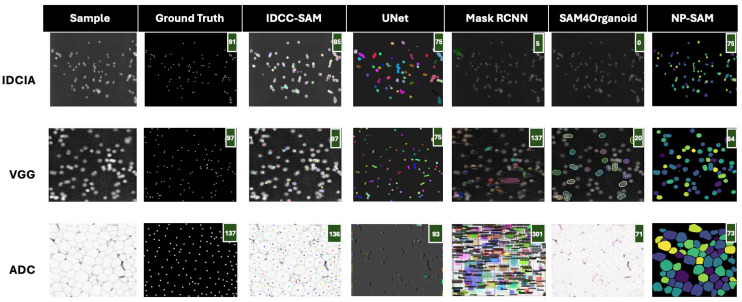
Qualitative results on test datasets—good examples. We show sample images, their corresponding human-annotated ground truths, and good examples of masks generated by IDCC-SAM. We also show the corresponding masks of the four (4) benchmark methods on these samples. Predicted counting results are shown at the top-right corner in the green background box. IDCC-SAM has the closest cell count to the ground truth, compared with the other models.

**Figure 5:**
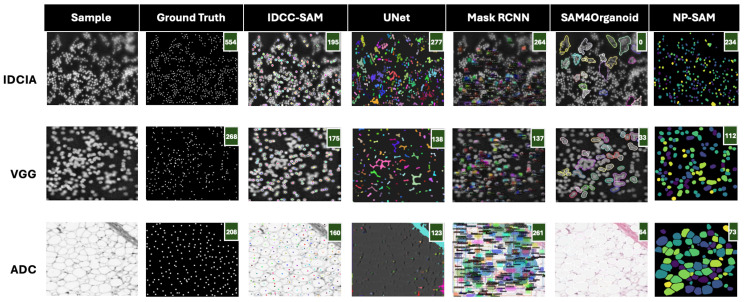
Qualitative results on test datasets—suboptimal examples. We show sample images, their corresponding human-annotated ground truths, and suboptimal examples of masks generated by IDCC-SAM. We also show the corresponding masks of the other four (4) benchmark methods on these samples. Predicted counting results are shown at the top-right corner in the green background box. Although these are suboptimal examples, IDCC-SAM still has the closest cell count to the ground truth, compared with the other benchmark models.

In Figure 4 and Figure 5, we present qualitative comparisons of IDCC-SAM and four benchmark methods (UNet, Mask RCNN, SAM4Organoid, and NP-SAM) on three datasets: IDCIA, VGG, and ADC. The performance is evaluated in terms of how closely the predicted cell counts match the ground truths, with predicted counts displayed in green in the top-right corner of each mask. A perfect alignment between ground truth and predictions is desirable, with lower deviations indicating better performance.

In Figure 4, IDCC-SAM achieves near-perfect performance on the VGG and ADC datasets. For example, in the VGG dataset, the ground truth shows 97 cells, and IDCC-SAM accurately predicts 97, outperforming UNet (75), Mask RCNN (75), and NP-SAM (64). Similarly, on the ADC dataset, IDCC-SAM predicts 136 cells, almost identical to the ground truth of 137, significantly outperforming SAM4Organoid, which predicts 301 cells—a gross overestimation. Even on the challenging IDCIA dataset, IDCC-SAM demonstrates its strengths, predicting 85 cells compared with the ground truth of 91, while Mask RCNN predicts only 75 cells.

In contrast, Figure 5 highlights suboptimal examples. On the IDCIA dataset, the ground truth is 554 cells, but IDCC-SAM predicts 195 cells, which, although lower, still surpasses SAM4Organoid’s complete failure with a prediction of 0 cells. Similarly, in the VGG dataset, IDCC-SAM predicts 175 cells against the ground truth of 268, while Mask RCNN and NP-SAM predict 138 and 112, respectively. For ADC, IDCC-SAM predicts 160 cells, closer to the ground truth of 208 than SAM4Organoid’s overestimation of 261. These examples illustrate IDCC-SAM’s general ability to provide closer predictions compared with the benchmark models, even in challenging cases.

**Figure 6:**
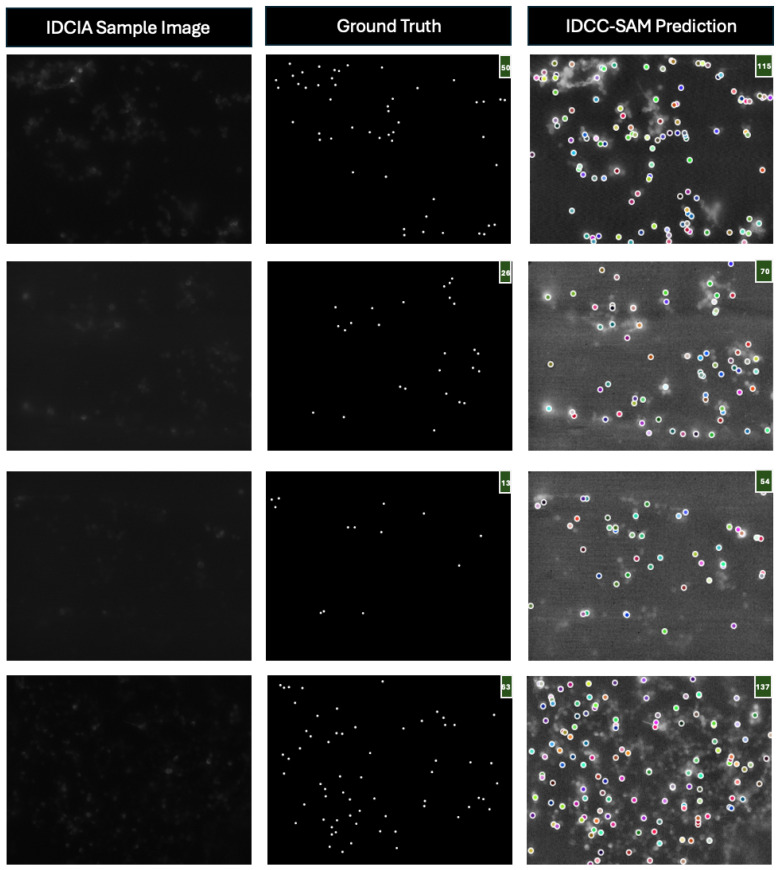
Examples of IDCC-SAM’s robustness in handling noisy ground truths. This figure shows sample IDCIA images, their corresponding human-annotated ground truths, and the predictions generated by IDCC-SAM. In several cases, IDCC-SAM’s predictions visually align more accurately with the original cellular structures compared with the ground truth annotations. This highlights IDCC-SAM’s ability to overcome inconsistencies in manual annotations, demonstrating its potential for improved precision and reliability in automated cell counting tasks.

Finally, Figure 6 highlights an important strength of IDCC-SAM: its ability to produce predictions that often align more closely with the original images than the human-annotated ground truths. For instance, in one example, IDCC-SAM predicts 70 cells, while the ground truth counts only 20, suggesting that the ground truth may be underestimating the true cell count. This demonstrates IDCC-SAM’s robustness in handling noisy images, as seen in the IDCIA dataset. These findings not only showcase IDCC-SAM’s potential to mitigate inconsistencies inherent in manual annotations, but also emphasize the importance of automated methods for ensuring more accurate and reproducible cell counting.

Overall, our result has shown that IDCC-SAM is good for standard cell counting, performing better than most of its supervised and zero-shot counterparts across three (3) different datasets. With some fine-tuning, its performance can be even better. Its ability to adapt to multiple datasets, immunocytochemistry staining techniques, and cellular image morphology, even under zero-shot settings, highlights its potential and suggests that IDCC-SAM could be a valuable tool for researchers and practitioners working with diverse cellular image datasets, particularly where manual annotation is challenging.

## 5. Conclusions

In this study, we introduced IDCC-SAM, a novel end-to-end pipeline that leverages the Segment Anything Model (SAM) for fully automatic cell counting in immunocytochemistry datasets. By employing a zero-shot approach, IDCC-SAM eliminates the need for labeled training data, offering significant improvements over both supervised and zero-shot benchmark methods. Our results demonstrate the effectiveness and adaptability of IDCC-SAM in three diverse datasets, showcasing its potential to advance the state-of-the-art in automated cell counting tasks.

This study:

- Provides a foundation for further advancements in automated cell counting methods, particularly in challenging and diverse imaging conditions.
- Stimulates the exploration of innovative fine-tuning strategies to enhance zero-shot performance in biomedical imaging tasks.

## 6. Future Work

While IDCC-SAM demonstrates strong generalizability and scalability, two (2) major limitations were identified during the evaluation:

- **Performance on high-density cell images:** IDCC-SAM struggles with accurately delineating overlapping cells in densely packed images, particularly in the IDCIA dataset.
- **Sensitivity to variability in staining techniques:** the diverse staining methods and cell morphologies in some datasets impact segmentation accuracy, highlighting the need for greater adaptability.

To address these limitations and further enhance IDCC-SAM, we propose the following future directions:

- **CLIP integration:** explore integrating CLIP (Contrastive Language–Image Pre-training) into IDCC-SAM to enhance multimodal understanding, improving segmentation and counting accuracy in diverse staining and morphological conditions.
- **Few-shot fine-tuning:** investigate the effectiveness of fine-tuning IDCC-SAM on a small subset of labeled data to improve its performance on high-density cell images and complex staining techniques.
- **Domain-adaptive fine-tuning:** develop a domain-adaptive encoder and decoder tailored for immunocytochemistry images to improve robustness and adaptability to varied staining methods and cell morphologies.
- **Dynamic patch sizes:** explore the use of dynamic patch sizes in the ViT encoder to enhance the model’s ability to capture both fine-grained and global contextual features in densely packed cellular images.
- **Integration with downstream tasks:** extend IDCC-SAM’s application to related tasks such as cell classification, clustering, and morphological analysis to evaluate its versatility beyond cell counting.

## Figures and Tables

**Table 1:** Summary of related works.

Method Category	Technique Description	Related Work	Key Advantages	Key Challenges
Image Processing-Based	Edge detection, thresholding, and morphological operations	[14,16,18]	Computationally efficient, simple, and effective for preprocessed images	Struggles with overlapping cells, noisy data, or inconsistent lighting
Density-Based Methods	Density estimation and clustering techniques	[20,21,22,23]	Handles varying densities, suitable for complex and dense images	Requires high computational resources and careful parameter tuning
Segmentation-Based Methods	Deep learning-based semantic segmentation (e.g., U-Net)	[24,25,26]	Highly accurate and scalable with deep learning models	Relies on large annotated datasets, which are expensive and time-consuming to create
Foundation Models	Pre-trained models for image segmentation and classification	[27,28,29]	Adaptable to new tasks with pre-trained capabilities	Tasks requiring extremely high precision may still need model fine-tuning and training

**Table 2:** Comparison of three (3) datasets used for evaluation of IDCC-SAM.

Dataset	Number of Images	Avg Cell Count Per Image
IDCIA	262	83 ± 104
VGG	200	174 ± 64
ADC	200	165 ± 44
Overall:	662	140 ± 70

**Table 3:** Average MAE and AAE of IDCC-SAM, evaluated on low-density (GT ≤ 100 cells) vs. high-density (GT > 100 cells) images. GT = ground truth.

Dataset	MAE (Low Density)	MAE (High Density)	AAE (Low Density)	AAE (High Density)
VGG	11	30	44%	23%
ADC	8	29	100%	23%
IDCIA	37	90	40%	14%

**Table 4:** Average MAE and AAE of of the five methods on VGG dataset, evaluated on low-density (GT ≤ 100 cells) vs. high-density (GT > 100 cells) images. GT = ground truth.

Model	MAE (GT ≤ 100)	MAE (GT > 100)	AAE (GT ≤ 100)	AAE (GT > 100)
UNet	17	68	22%	0%
Mask RCNN	71	36	0%	16%
SAM4Organoid	77	155	0%	0%
NP-SAM	19	78	11%	0%
IDCC-SAM (Ours)	11	30	44%	23%

**Table 5:** Average MAE and AAE of of the five methods on ADC dataset, evaluated on low-density (GT ≤ 100 cells) vs. high-density (GT > 100 cells) images. GT = ground truth.

Model	MAE (GT ≤ 100)	MAE (GT > 100)	AAE (GT ≤ 100)	AAE (GT > 100)
UNet	50	57	0%	10%
Mask RCNN	150	129	0%	0%
SAM4Organoid	53	101	0%	0%
NP-SAM	49	91	0%	0%
IDCC-SAM (Ours)	8	29	100%	23%

**Table 6:** Average MAE and AAE of of the five methods on IDCIA dataset, evaluated on low-density (GT ≤ 100 cells) vs. high-density (GT > 100 cells) images. GT = ground truth.

Model	MAE (GT ≤ 100)	MAE (GT > 100)	AAE (GT ≤ 100)	AAE (GT > 100)
UNet	27	84	40%	11%
Mask RCNN	33	64	36%	18%
SAM4Organoid	39	194	25%	0%
NP-SAM	35	77	31%	11%
IDCC-SAM (Ours)	37	90	40%	14%

**Table 7:** Overall comparison of Avg. MAE and AAE of the five methods on all datasets.

	VGG		ADC		IDCIA	
Model	MAE	AAE	MAE	AAE	MAE	AAE
UNet	57	5%	57	10%	43	32%
Mask RCNN	44	13%	130	0%	48	24%
SAM4Organoid	138	0%	99	0%	83	17%
NP-SAM	65	3%	89	0%	47	25%
IDCC-SAM (Ours)	26	28%	28	26%	52	33%

## Data Availability

Our codes, datasets, results, and instructions to reproduce our results is publicly available at https://github.com/DickersonLab/IDCC-SAM.

## Supplementary Materials

The following supporting information can be downloaded at: https://www.mdpi.com/article/10.3390/bioengineering12020184/s1, Supplementary File: IDCC-SAM Supporting Information File.
