# Supplementary material for: IDCC-SAM: A Zero-Shot Approach for Cell Counting in Immunocytochemistry Dataset Using the Segment Anything Model
Source: Bioengineering (Basel). 2025 Feb 14;12(2):184. doi: 10.3390/bioengineering12020184 (PMC11851800; doi:10.3390/bioengineering12020184)
Supplement: Supplementary file 1 [file bioengineering-12-00184-s001.zip › bioengineering-3455889-supplementary.pdf]

Supplementary Information for:

IDCC-SAM: A Zero-Shot Approach for Cell Counting in Immunocytochemistry Dataset Using the Segment Anything Model

Samuel Fanijo[1,*], Ali Jannesari[1], Julie Dickerson[2,*]

1 Department of Computer Science, Iowa State University, USA

2 Department of Electrical and Computer Engineering, Iowa State University, USA

*Corresponding Authors

Email: sfanijo@iastate.edu (SF), julied@iastate.edu (JD)

Experimental Setup for fine-tuned baseline models

UNet

Training Data: The datasets used in this study are public and published in another paper and have been appropriately referenced and described in the manuscript. The preprocessed version for this study is available at `https://github.com/DickersonLab/IDCC-SAM`. The dataset was split into training and validation sets using a 75-25 ratio.

Hyperparameters: We used default parameters for fine-tuning to ensure fairness. As common to binary segmentation tasks in UNet, we utilized the binary cross-entropy loss function and the Adam optimizer. All details are provided in our code implementation and instructions (available at `https://github.com/DickersonLab/IDCC-SAM`).

Experimental Environment: The experiments were conducted using TensorFlow and

Keras deep learning frameworks. We trained the model on a single TensorFlow GPU on Google Colab for 50 Epochs.

Mask RCNN

Training Data: The datasets used in this study are public and published in another paper and have been appropriately referenced and described in the manuscript. The preprocessed version for this study is available at `https://github.com/DickersonLab/IDCC-SAM`. Furthermore, the dataset was converted into a coco format relevant to the model architecture, split into training and validation sets using a 75-25 ratio.

Hyperparameters: The Mask RCNN comes with a model configuration file containing a set of default training parameters. We used them as they were for fine-tuning to ensure fairness. The model details are provided in our code implementation and instructions (available at `https://github.com/DickersonLab/IDCC-SAM`).

Experimental Environment: The experiments were conducted using TensorFlow and Keras deep learning frameworks. We trained the model on a single TensorFlow GPU on Google Colab for 500 maximum iterations.

NOTE: As SAM4Organoid and NP-SAM are both zero-shot baselines, no additional experimental setup is required for them.

Similarly, as our method, IDCC-SAM, is a zero-shot pipeline, no additional experimental setup is required.
